# Premature mitotic entry induced by ATR inhibition potentiates olaparib inhibition‐mediated genomic instability, inflammatory signaling, and cytotoxicity in BRCA2‐deficient cancer cells

**DOI:** 10.1002/1878-0261.12573

**Published:** 2019-10-21

**Authors:** Pepijn M. Schoonen, Yannick P. Kok, Elles Wierenga, Bjorn Bakker, Floris Foijer, Diana C. J. Spierings, Marcel A. T. M. van Vugt

**Affiliations:** ^1^ Department of Medical Oncology University Medical Center Groningen University of Groningen The Netherlands; ^2^ European Institute for the Biology of Ageing (ERIBA) University Medical Center Groningen University of Groningen The Netherlands

**Keywords:** cell cycle, cGAS/STING, checkpoint, genomic instability, mitosis, single‐cell sequencing

## Abstract

Poly(ADP‐ribose) polymerase (PARP) inhibitors are selectively cytotoxic in cancer cells with defects in homologous recombination (HR) (e.g., due to *BRCA1/2* mutations). However, not all HR‐deficient tumors efficiently respond to PARP inhibition and often acquire resistance. It is therefore important to uncover how PARP inhibitors induce cytotoxicity and develop combination strategies to potentiate PARP inhibitor efficacy in HR‐deficient tumors. In this study, we found that forced mitotic entry upon ATR inhibition potentiates cytotoxic effects of PARP inhibition using olaparib in BRCA2‐depleted and *Brca2* knockout cancer cell line models. Single DNA fiber analysis showed that ATR inhibition does not exacerbate replication fork degradation. Instead, we find ATR inhibitors accelerate mitotic entry, resulting in the formation of chromatin bridges and lagging chromosomes. Furthermore, using genome‐wide single‐cell sequencing, we show that ATR inhibition enhances genomic instability of olaparib‐treated BRCA2‐depleted cells. Inhibition of CDK1 to delay mitotic entry mitigated mitotic aberrancies and genomic instability upon ATR inhibition, underscoring the role of ATR in coordinating proper cell cycle timing in situations of DNA damage. Additionally, we show that olaparib treatment leads to increased numbers of micronuclei, which is accompanied by a cGAS/STING‐associated inflammatory response in BRCA2‐deficient cells. ATR inhibition further increased the numbers of cGAS‐positive micronuclei and the extent of cytokine production in olaparib‐treated BRCA2‐deficient cancer cells. Altogether, we show that ATR inhibition induces premature mitotic entry and mediates synergistic cytotoxicity with PARP inhibition in HR‐deficient cancer cells, which involves enhanced genomic instability and inflammatory signaling.

**Abbreviations**
53BP1: TP53‐binding protein‐1
ATR: ataxia telangiectasia and Rad3‐related
BRCA1: breast cancer 1, early onset
BRCA2: breast cancer 2, early onset
CCL5: C‐C motif chemokine‐5
CDK1: cyclin‐dependent kinase‐1
cGAS: cyclic GMP‐AMP synthase
CI: combination index
DSB: double‐stranded break
EMI1: early mitotic inhibitor‐1
FANCD2: Fanconi anemia group D2
GAPDH: glyceraldehyde‐3‐phosphate dehydrogenase
Hprt: hypoxanthine‐guanine phosphoribosyltransferase
HR: homologous recombination
HU: hydroxyurea
iBAC: infectious bacterial artificial chromosome
IRF3: interferon regulatory factor‐3
MRE11: meiotic recombination‐11
PARP: poly(ADP‐ribose) polymerase
PD‐1: programmed cell death protein‐1
RT‐PCR: reverse transcription polymerase chain reaction
SCR: scrambled
scWGS: single‐cell whole‐genome sequencing
siRNA: small interference RNA
SSB: single‐stranded DNA break
STING: stimulator of interferon genes

## 1 Introduction

BRCA1 (breast cancer, early onset 1) and BRCA2 (breast cancer, early onset 2) are essential components of the homologous recombination (HR) DNA repair machinery, which repairs toxic DNA double‐stranded breaks (DSBs) (Thompson and Schild, 2001). Loss of BRCA1 or BRCA2 results in genomic instability, underscoring the essential role for HR in genome maintenance (Evers *et al.*, 2008). Importantly, mutations in HR genes, including in *BRCA2*, result in a highly increased lifetime risk to develop breast and ovarian cancer (Wooster *et al.*, 1994). Interestingly, due to their DNA repair defect, BRCA‐mutant tumors show increased sensitivity to certain DNA‐damaging agents, including platinum‐based chemotherapeutics. Additionally, BRCA‐mutant tumors are selectively sensitive to inhibition of poly(ADP‐ribose) polymerase (PARP) (Bryant *et al.*, 2005; Farmer *et al.*, 2005), which has led to the successful implementation of PARP inhibitors as a treatment strategy for *BRCA1* or *BRCA2* mutant tumors (Audeh *et al.*, 2010; Tutt *et al.*, 2010).

Poly(ADP‐ribose) polymerase inhibition results in DNA lesions during DNA replication through multiple mechanisms. PARP1 is involved in repair of single‐strand DNA breaks (SSBs). SSBs can be converted into DNA DSBs during replication, when they remain unrepaired due to PARP inhibition (Bryant *et al.*, 2005; Farmer *et al.*, 2005). More recently, the ability of PARP inhibitors to kill HR‐deficient cells was shown to be related to the capacity of PARP inhibitor to trap PARP molecules onto DNA. These trapped PARP molecules subsequently lead to stalling and collapse of replication forks, which creates a dependency on functional HR for cellular survival (Murai *et al.*, 2012). In addition, PARP1 was shown to restrain replication fork speed, which underlies disturbed replication kinetics upon PARP inhibition (Maya‐Mendoza *et al.*, 2018).

Cancer cells lacking functional BRCA2 are defective in protecting nascent DNA from degradation at stalled replication forks (Schlacher *et al.*, 2011; Ying *et al.*, 2012) and cannot properly repair the DSBs that result from forks collapse (Moynahan *et al.*, 2001). Typically, accumulation of DSBs leads to activation of the G2/M cell cycle checkpoint, which prevents entry into mitosis (Löbrich and Jeggo, 2007). Whether replication‐born DNA lesions efficiently trigger a G2/M checkpoint response remains unclear. Accumulating evidence shows that unresolved replication lesions do not necessarily block mitotic entry and are transmitted into mitosis, leading to mitotic aberrancies (Chan *et al.*, 2018, 2009; Naim *et al.*, 2013; Schoonen *et al.*, 2017).

Indeed, PARP inhibition in BRCA1 or BRCA2‐defective cancer cells leads to increased levels of mitotic aberrancies including chromatin bridges and micronuclei (Chan *et al.*, 2018; Feng and Jasin, 2017; Laulier *et al.*, 2011). Notably, the presence of such mitotic aberrancies upon PARP inhibition was strongly associated with PARP inhibitor‐induced cytotoxicity (Chan *et al.*, 2018). Interestingly, the micronuclei that originate as a consequence of defective DNA repair, including BRCA2 inactivation, were shown to activate a cGAS/STING (cyclic GMP‐AMP synthase/stimulator of interferon genes)‐dependent interferon response (Bakhoum *et al.*, 2018; Heijink *et al.*, 2019; MacKenzie *et al.*, 2017). Indeed, treatment of *Brca1*‐defective ovarian tumors with a PARP inhibitor was shown to trigger cGAS/STING signaling and thereby sensitize these tumors to PD‐1 blockade treatment (Ding *et al.*, 2018).

Although HR‐defective cancer cells show profound sensitivity to PARP inhibition, multiple mechanisms of acquired resistance have been described, including genetic reversion of the *BRCA1* or *BRCA2* mutations (Edwards *et al.*, 2008; Norquist *et al.*, 2011; Sakai *et al.*, 2008; Swisher *et al.*, 2008), inactivation of the 53BP1/shieldin pathway (Bunting *et al.*, 2010; Noordermeer *et al.*, 2018), downregulation of EMI1 (early mitotic inhibitor‐1) (Marzio *et al.*, 2018; Schoonen *et al.*, 2017), or alterations at the levels of PARP1/2 abundance or activity (Gogola *et al.*, 2018; Henneman *et al.*, 2015; Liu *et al.*, 2009; Murai *et al.*, 2012; Oplustilova *et al.*, 2012; Pettitt *et al.*, 2018). It is therefore pivotal to find successful combination strategies to potentiate PARP inhibitor efficacy.

A potentially effective strategy to potentiate PARP inhibitor‐mediated cytotoxicity would be to target the G2/M cell cycle checkpoint to force mitotic entry in the presence of DNA lesions. Indeed, it was previously demonstrated that progression through mitosis promotes PARP inhibitor cytotoxicity in HR‐deficient cells (Schoonen *et al.*, 2017) and that inactivation of cell cycle checkpoint kinases, including ATR (ataxia telangiectasia and Rad3‐related), could potentiate the cytotoxicity of PARP inhibition (Kim *et al.*, 2017; Michelena *et al.*, 2018; Schoonen *et al.*, 2017). Because cell cycle checkpoint kinases have functions beyond regulating the G2/M cell cycle checkpoint (Byun *et al.*, 2005; Domínguez‐Kelly *et al.*, 2011; Matthew and Newport, 1998), in this study we investigated the role of ATR inhibition in potentiating the effects of the PARP inhibitor olaparib.

## 2 Materials and methods

### 2.1 Cell culture

The HeLa human cervical cancer cell line was obtained from ATCC (Manassas, VA, USA) (#CCL2). Human retinal epithelium RPE‐1 cells were obtained from Bob Weinberg (MIT, Cambridge, MA, USA). HeLa and RPE‐1 cells were cultured in Dulbecco's modified Eagle's medium (DMEM), supplemented with 10% FBS, 50 units·mL^−1^ penicillin, 50 µg·mL^−1^ streptomycin, and 5 µg·mL^−1^ insulin (Sigma, Saint Louis, MO, USA), in a humidified incubator supplied with 5% CO_2_ at 37 °C. Cell lines were verified by STR profiling (BaseClear, Leiden, the Netherlands). The KB2P1.21 cell line was established from a mammary tumor from *K14cre;Brca2^F11/F11^;p53^F2‐10/F2‐10^* mice as described previously (Evers *et al.*, 2008). The KB2P1.21R1 cell line was created by the stable introduction of an iBAC, containing the full‐length mouse *Brca2* gene, into the KB2P1.21 cell line (Evers *et al.*, 2008). The *K14cre;Brca2^wt/wt^;p53^F2‐10/F2‐10^* cell line KP3.33 was obtained from Jos Jonkers (NKI, Amsterdam, the Netherlands). All murine cell lines were cultured in DMEM/F‐12 medium, supplemented with 10% FBS, 50 units·mL^−1^ penicillin, 50 µg·mL^−1^ streptomycin, 5 µg·mL^−1^ insulin (Sigma), 5 ng·mL^−1^ epidermal growth factor (Life Technologies, Carlsbad, CA, USA), and 5 ng·mL^−1^ cholera toxin (Gentaur, Kampenhout, Belgium), at 37 °C under hypoxic conditions (1% O_2_, 5% CO_2_).

### 2.2 MTT assays

HeLa, KB2P1.21, and KB2P1.21R1 tumor cell lines were plated in 96‐well plates. HeLa were plated at 2000 cells per well, and KB2P1.21 and KB2P1.21R1 were plated at 1200 cells per well. Cells were first grown for 3 or 24 h and were subsequently treated with the indicated concentrations of olaparib and VE‐821 for 3 days. Methyl‐thiazol tetrazolium (MTT) was added to cells at a concentration of 5 mg·mL^−1^ for 4 h, after which culture medium was removed and formazan crystals were dissolved in DMSO. Absorbance values were determined using a Bio‐Rad (Hercules, CA, USA) Benchmark III Biorad microtiter spectrophotometer at a wavelength of 520 nm. Viability was determined by comparing absorbance values to those of DMSO‐treated cells. Experiment was performed in triplicate. Graphs show representative experiments, which were performed at least twice.

### 2.3 RNA interference

For siRNA (small interference RNA) transfection, siRNAs (Ambion Stealth RNAi; Thermo Fisher, Waltham, MA, USA) targeting *BRCA2* (sequence 1: #HSS186121; and sequence 2: sequence #HSS101095) or a siSCR (scrambled) control sequence (sequence #12935300) were used at a final concentration of 40 nm. Transfections were performed with Oligofectamine (Invitrogen, Carlsbad, CA, USA) according to the manufacturer’s guidelines.

### 2.4 Western blotting

Cell lysis was performed using Mammalian Protein Extraction Reagent (MPER; Thermo Scientific), supplemented with protease inhibitor and phosphatase inhibitor (Thermo Scientific). Protein concentrations were measured using a Bradford assay. Next, proteins were separated by SDS/PAGE and transferred to polyvinylidene fluoride (PVDF; Immobilon, Merck, Burlington, MA, USA) membranes and blocked in 5% skimmed milk (Sigma) in Tris‐buffered saline (TBS) containing 0.05% Tween‐20 (Sigma). Immunodetection was performed with antibodies directed against BRCA2 (Calbiochem, Merck, Burlington, MA, USA; #OP95), PAR (Trevigen, Gaithersburg, MD, USA; #4336‐BPC‐100), phospho‐ATR (thr1898; Millipore, Burlington, MA, USA; #ABE462), STING (1 : 1000; Cell Signaling, Danvers, MA, USA, #13647), cGAS (1 : 1000; Cell Signaling; #15102S) IRF3 (1 : 1000; Cell Signaling; #4302), p‐IRF3 (1 : 100; Cell Signaling; #29047), and β‐actin (MP Biomedicals, Santa Ana, CA, USA; #69100). Horseradish peroxidase (HRP)‐conjugated secondary antibodies (DAKO, Glostrup, Denmark) were used for visualization using chemiluminescence (Lumi‐Light; Roche Diagnostics, Basel, Switzerland) on a Bio‐Rad bioluminescence device, equipped with quantity one/chemidoc xrs software (Bio‐Rad).

### 2.5 Immunofluorescence microscopy

HeLa, KB2P1.21, and KB2P21R1 cells were seeded on glass coverslips in 6‐well plates. When indicated, HeLa cells were transfected with siRNAs for 48 h, of which the final 24 h included treatment with olaparib (0.5 µm) and/or VE‐821 (1 µm) for 24 h as indicated. For DNA bridge, micronuclei, cGAS (Cell Signaling; #15102), or RAD51 (GeneTex, Irvine, CA, USA; GTX7023) staining, cells were fixed using 4% formaldehyde in PBS and subsequently permeabilized for 5 min in PBS with 0.1% Triton X‐100. For FANCD2 (Novusbio, Centennial, CO, USA; NB100‐182) and γ‐H2AX (Millipore; 05‐636) staining, cells were treated for 60 s with PEM [100 mm PIPES (pH 6.9), 1 mm MgCl_2_, and 10 mm EGTA]. Next, cells were simultaneously fixed and permeabilized [20 mm PIPES (pH 6.8), 0.2% Triton X‐100, 1 mm MgCl2, 10 mm EGTA, 4% paraformaldehyde] for 10 min at room temperature. Cells were then incubated with corresponding Alexa‐488‐ or Alexa‐647‐conjugated secondary antibodies and counterstained with DAPI (Sigma). For analysis of DNA damage response components, prophase and pro‐metaphase cells were included for scoring, specifically mitotic cells with condensed chromosome, but prior to metaphase alignment. For analysis of chromatin bridges and lagging chromosomes, anaphase and telophase cells were distinguished based on α‐tubulin staining (Cell Signaling; #2125). Images were acquired on a Leica DM6000B microscope using a 63× immersion objective (PL S‐APO, numerical aperture: 1.30) with las‐af software (Leica, Wetzlar, Germany).

### 2.6 Cytokine analysis

To analyze excreted CCL5 levels, KB2P1.21 and KB2P1.21R1 tumor cell lines cells were treated with 0.5 µm olaparib and/or 1 µm VE‐821. Culture media were collected after 24 h. Subsequently, CCL5 concentrations were determined using the mouse CCL5 ELISA kit (R&D Systems, Minneapolis, MN, USA #MMR00) according to the manufacturer’s protocol.

### 2.7 DNA fiber analysis

For DNA fiber analysis, HeLa or RPE‐1 cells were pulse‐labeled with CIdU (25 µm) for 60 min followed by IdU (250 µm) for 60 min when indicated. Next, cells were washed with warm medium and incubated with hydroxyurea (HU, 5 mm) for 5 h. Cells were then trypsinized and lysed in lysis buffer [0.5% SDS, 200 mm Tris (pH 7.4), 50 mm EDTA] on tilted microscopy slides. Following DNA spreading, slides were air‐dried and fixed in methanol/acetic acid (3 : 1) for 10 min. For immunolabeling, slides with DNA spreads were incubated in 2.5 m HCl for 1.5 h. Primary antibodies used were rat anti‐BrdU (1 : 1000; Abcam, Cambridge, UK; Ab6326) for CldU detection and mouse anti‐BrdU (1 : 500; ExBio, Vestec, Czech Republic; 11‐286‐C100) for IdU detection. Secondary antibodies were incubated for 1 h and were then further incubated with Alexa Fluor 488‐ or 647‐conjugated secondary antibodies (1 : 500) for 1.5 h. Images were acquired on a Leica DM‐6000RXA fluorescence microscope, equipped with Leica Application Suite software. The lengths of CIdU and IdU tracks were measured blindly using imagej software (NIH, Bethesda, MD, USA). Two‐sided Mann–Whitney tests with 95% confidence intervals were used for statistical analysis.

### 2.8 Cell cycle analysis

Cells were synchronized at G1/S phase using a double‐thymidine block. Specifically, cells were treated with thymidine (2 mm; Sigma) for 17 h, washed twice with prewarmed PBS, and incubated in prewarmed warm medium for 9 h. Subsequently, cells were again incubated in thymidine for 17 h, after which cells were washed with PBS and released in prewarmed medium containing olaparib (1 µm), VE‐821 (1 µm), or both, and collected at the indicated time points. When indicated, cells were trapped in mitosis using a 16‐h incubation with nocodazole (100 ng·mL^−1^; Sigma). Cells were then fixed in ice‐cold ethanol (70%) for at least 16 h and were stained with phospho‐histone‐H3 (Ser10; Cell Signaling; #9701, 1 : 50) in combination with Alexa‐488‐conjugated secondary antibodies (1 : 200). DNA staining was performed using propidium iodide in the presence of RNase. At least 10 000 events per sample were analyzed on a FACSCalibur (Becton Dickinson, Franklin Lakes, NJ, USA). Data were analyzed using flowjo software (Becton Dickinson).

### 2.9 RT‐PCR

RNA was isolated from KB2P1.21R1, KB2P1.21, and KP3.33 using Qiagen RNeasy kit (Qiagen, Hilden, Germany), and a total of 0.5µg RNA was used as input for cDNA preparation (iScript™). Subsequently, 16 ng cDNA was used for a quantitative PCR using the SYBR Green PCR Master Mix (Applied Biosystems, Foster City, CA, USA). Hypoxanthine‐guanine phosphoribosyltransferase (Hprt) and glyceraldehyde‐3‐phosphate dehydrogenase (GAPDH) were used as control. The following primers were used for Brca2: exon 10–11 forward: gaagcaagtgcttttgaag and reverse: cagaagaatctggtatacctg; and exon 18–19 forward: ctcctgatgcctgtgcacc and reverse: cacgaaagaaccccagcct.

### 2.10 Single‐cell whole‐genome analysis

HeLa cells were incubated in mild lysis buffer, and single G1 nuclei were sorted into 96‐well plates, using a Hoechst/propidium iodide double staining. Illumina‐based library preparation was performed as described previously (van den Bos *et al.*, 2016), in an automated fashion using a Bravo automated liquid handling platform (Agilent Technologies, Santa Clara, CA, USA). Single‐cell libraries were pooled and sequenced on an Illumina NextSeq 500 sequencer (Illumina, San Diego, CA, USA). Sequencing data were analyzed using aneufinder software as described previously (Bakker *et al.*, 2016). Per sample and per bin, the modal copy number state of siSCR control‐treated cells was determined, and bins that deviated from the modal copy number state were identified. The genomic instability scores were assessed per cell, by determining the fraction of bins that deviate from the modal copy number for that sample. All sequencing data have been deposited at the European Nucleotide Archive under accession no. PRJEB31290.

## 3 Results

### 3.1 PARP inhibitor treatment induces a concentration‐dependent G2 arrest

**Figure 1:**
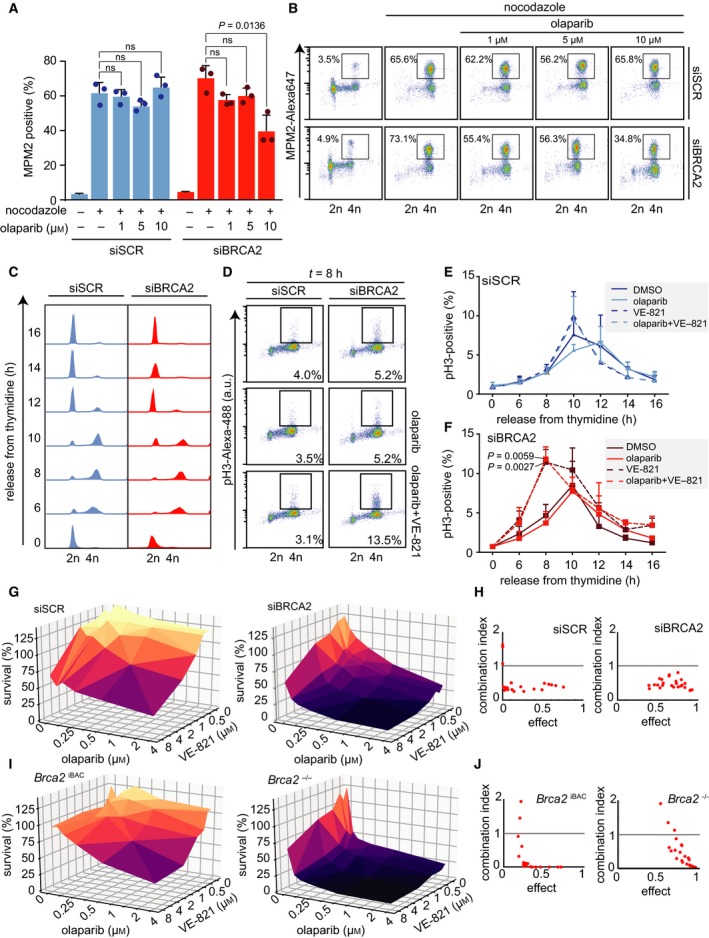
ATR inhibition induces premature mitotic entry and is synergistically cytotoxic with PARP inhibition. (A) HeLa cells were transfected with control (siSCR, #12935300) or BRCA2 (‘siBRCA2 #1’, #HSS186121) siRNAs for 24 h and subsequently treated with DMSO and/or olaparib (1, 5, or 10 µm) 24 h prior to harvesting. Next, cells were treated with nocodazole (250 ng·mL^−1^) for 18 h. DNA content (propidium iodine) and MPM2‐Alexa‐647 were assessed by flow cytometry on a Becton Dickinson FACSCalibur (Becton Dickinson). A minimum of 10 000 events were analyzed per sample. Averages and standard deviations of three biological replicates are shown (*n* = 3). *P* values were calculated using two‐tailed Student’s *t*‐test. (B) Representative MPM2‐Alexa‐647 plots are shown of HeLa cells treated as described in panel A. (C, D) HeLa cells were transfected with control or BRCA2 siRNAs for 24 h and subsequently incubated with thymidine (2 mm) for 17 h. Cells were then released for 9 h in prewarmed growth media and again treated for 17 h with thymidine prior to release in growth media supplemented with DMSO, olaparib (1 µm), and/or VE‐821 (1 µm). Cells were harvested at the indicated time points for flow cytometry analysis. DNA content (propidium iodine) and pH3‐Ser10/Alexa‐488 were assessed by flow cytometry on a Becton Dickinson FACSCalibur (Becton Dickinson). A minimum of 10 000 events were analyzed per sample. Representative DNA plots are shown in panel C. Representative phospho‐Ser10‐histone‐H3 plots are shown in panel D. (E, F) Quantification of results of panel D. Averages and standard deviations of three biological replicates are shown. *P* values were calculated using a two‐tailed Student’s *t*‐test. (G) HeLa cells were transfected with control siRNAs or siRNAs targeting BRCA2 and were treated with the indicated concentrations of olaparib and/or ATR inhibitor VE‐821. Methyl‐thiazol tetrazolium (MTT, 0.5 mg·mL^−1^) was added for 4 h, and viability was assessed by colorimetric measurement. (H) Combination indices (CI) for HeLa cell treated as described in panel g were determined using compusyn software (Compusyn Inc., New York, NY, USA). (I) KB2P1.21 (‘*Brca2* ^−/−^’) and KB2P1.21R1 (‘*Brca2* ^iBAC^’) cells were treated and analyzed as described in panel g. (J) CI for treatments in *Brca2* ^iBAC^ and *Brca2* ^−/−^ cells were determined using compusyn software.

DNA lesions induced by PARP inhibitor treatment can trigger a G2 cell cycle arrest (Jelinic and Levine, 2014; Maya‐Mendoza *et al.*, 2018; Prasad *et al.*, 2017; Ray Chaudhuri *et al.*, 2016; Rein *et al.*, 2015). To investigate the extent to which PARP inhibition induces a G2 cell cycle arrest, BRCA2‐depleted HeLa cells were treated with increasing amounts of the PARP inhibitor olaparib. Next, cells were trapped in mitosis using the microtubule poison nocodazole, and the percentages of cells in G2 and mitosis were quantified using the mitotic markers MPM2 and phospho‐H3‐Ser10 (Fig. 1A,B and Fig. S1A). Interestingly, increasing concentrations of olaparib decreased the mitotic population in BRCA2‐depleted cells, but not in control cells (Fig. 1A and Fig. S1A). These data indicate that PARP inhibition dose‐dependently provokes a G2 arrest in BRCA2‐depleted cells. Next, we wanted to determine whether PARP inhibition can also provoke a G2‐arrest in more clinically relevant HR models. To this end, we used K14cre;*Brca2* ^del/del^;*p53* ^del/del^ mouse mammary tumor cells (further denoted as *Brca2* ^−/−^), together with an isogenic line in which BRCA2 expression was reconstituted using an infectious bacterial artificial chromosome (iBAC) containing the mouse *Brca2* gene (denoted as *Brca2* ^iBAC^) (Evers *et al.*, 2008, 2010). RT‐PCR analysis showed similar expression levels of *Brca2* in *Brca2* ^iBAC^ cells compared to wild‐type control cells (denoted as Brca2^+/+^), while *Brca2* ^−/−^ cells did not express transcripts containing *Brca2* exons 10–11 (Fig. S1B). Additionally, *Brca2* ^−/−^ cells failed to form irradiation‐induced Rad51 foci, which were rescued in *Brca2* ^iBAC^ cells, indicative of HR deficiency and restoration, respectively (Fig. S1C). Importantly, PARP inhibitor treatment resulted in decreased percentages of mitotic cells, in both *Brca2* ^−/−^ and *Brca2* ^iBAC^ cells (Fig. S1D). Combined, these data indicate that PARP inhibitor treatment induces delayed G2/M progression in a dose‐dependent fashion.

To reveal more subtle effects of PARP inhibitor treatment on cell cycle progression, BRCA2‐depleted HeLa cells were synchronized using a double‐thymidine block (Fig. 1C). Although BRCA2 depletion itself did not induce a detectable difference in cell cycle progression (Fig. 1C), addition of PARP inhibitor at the time of release from thymidine resulted in a minor but reproducible delay in mitotic entry (Fig. 1D–F). Notably, treatment with 1 µm olaparib resulted in a minor G2 delay, and treatment with a high olaparib concentration (10 µm) triggered a robust G2‐arrest (Fig. S1E,F).

To test the involvement of G2/M checkpoint activation in the PARP inhibitor‐induced G2 delay, we focused on the ATR cell cycle checkpoint kinase. Previously, ATR was shown to delay mitotic entry in response to DNA damage (Brown and Baltimore, 2003), as well as during unperturbed cell cycle (Lemmens *et al.*, 2018; Saldivar *et al.*, 2018). In line with these reports, we observed accelerated entry into mitosis upon ATR inhibition using VE‐821 in BRCA2‐depleted cells (Reaper *et al.*, 2011), either alone (DMSO versus VE‐821, *P* = 0.0059) or when combined with PARP inhibition (olaparib versus combined olaparib/VE‐821, *P* = 0.0027) (Fig. 1E,F and Fig. S1E,F). These data indicate that ATR inhibition can be utilized to promote premature mitotic entry of cells treated with PARP inhibitor.

### 3.2 PARP inhibition and ATR inhibition synergistically induce cancer cell killing

To investigate whether accelerated mitotic entry upon combined ATR and PARP inhibition results in increased cytotoxicity, cells were treated with increasing concentrations of olaparib and/or VE‐821. In line with earlier findings, PARP inhibition efficiently reduced cell viability in BRCA2‐depleted HeLa cells and *Brca2* ^−/−^ cells, whereas control cells were largely insensitive to PARP inhibition (Fig. 1G–J and Fig. S1G,H). When PARP inhibitor treatment was combined with ATR inhibition, synergistic loss of viability was observed in HR‐proficient cells, which underscores a requirement for ATR in HR (Wang *et al.*, 2004). Importantly, addition of ATR inhibition increased the sensitivity of HR‐deficient cells to PARP inhibition, which is in line with previous observations (Kim *et al.*, 2017; Fig. 1G–J and Fig. S1G,H). Combined ATR and PARP inhibition was synergistic at the majority of data points, as judged by combination index (CI) scores lower than 1 (Fig. 1H,J and Fig. S1H). Of note, only at the lowest drug concentrations CI index values were higher than 1, which can be explained by the absence of cytotoxic effects at these conditions. Combined, these data indicate that ATR inhibition and PARP inhibition are synergistically toxic.

### 3.3 ATR inhibition promotes mitotic entry in the presence of DNA damage

We next investigated mechanisms that could underlie the observed cytotoxic effects of combined ATR and PARP inhibition. In line with an ATR‐dependent cell cycle delay in BRCA2‐depleted cells (Fig. 1), we found that PARP inhibition leads to ATR activation in BRCA2‐depleted cells, as assessed by ATR autophosphorylation at Thr‐1989 (Fig. S2A). In these experiments, PARP was efficiently inhibited as judged by a near‐complete loss of PARylation (Fig. S2A).

**Figure 2:**
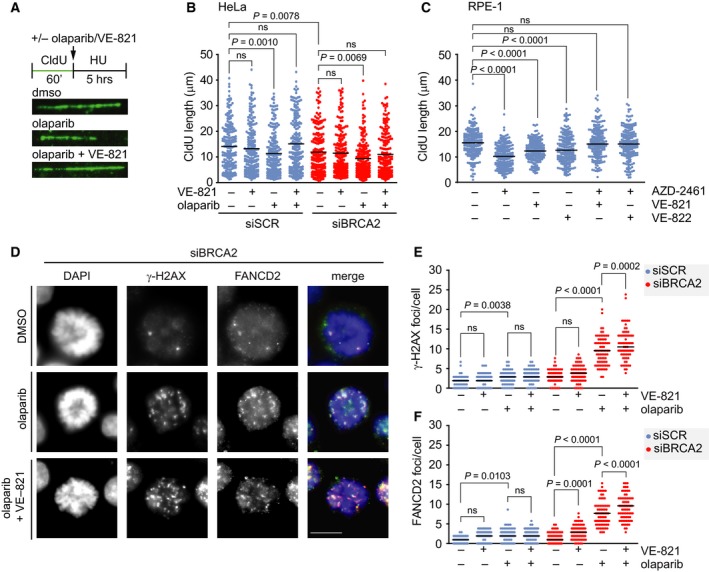
Combined ATR and PARP inhibition increases the amount of DNA damage in mitotic cells. (A) A schematic representation of treatment is shown. HeLa cells were transfected with control (siSCR) or BRCA2 (siBRCA2 #1) siRNA for 24 h and pulse‐labeled with CIdU for 60 min and were then treated with HU (5 mm), olaparib (5 μm), and/or VE‐821 (5 μm), as indicated, for 5 h. Cells were then lysed, and DNA was spread into single fibers. Representative immunofluorescence images of CldU tracks are shown. (B) CldU track length of cells from panel a was determined for 200 fibers per condition. *P* values were calculated using a two‐tailed Mann–Whitney test. (C) RPE‐1 cells were pulse‐labeled with CIdU for 60 min and were then treated with HU (5 mm), AZD‐2461 (1 μm), and/or VE‐821 (5 μm) or VE‐822 (1 μm), as indicated, for 3 h and analyzed as for panel B. *P* values were calculated using a two‐tailed Mann–Whitney test. (D) HeLa cells were transfected with control or BRCA2 siRNA for 24 h and were treated with olaparib (0.5 µm) and/or VE‐821 (1 µm). Cells were fixed and stained for γ‐H2AX (red) and FANCD2 (green) and counterstained with DAPI (blue). Representative immunofluorescence images are shown. (E, F) Numbers of γ‐H2AX foci (panel E) and FANCD2 foci (panel F) per mitotic nucleus were analyzed (*n* = 75 cells per condition). *P* values were calculated using a two‐tailed Mann–Whitney test. Throughout the figure, ‘ns’ indicates not significant. Scale bar represents 10 µm.

Next, we tested whether combined ATR and PARP inhibition exacerbated the degradation of stalled replication forks, since ATR inhibition was previously shown to destabilize stalled replication forks in PARP inhibitor‐resistant cells (Yazinski *et al.*, 2017). To this end, BRCA2‐depleted HeLa cells were incubated with the thymidine analog CldU to label nascent DNA at replication forks and were subsequently exposed to hydroxyurea (HU) to stall replication (Fig. 2A). In line with expectations, either PARP inhibition or BRCA2 depletion resulted in substantial degradation of nascent DNA at HU‐stalled forks (Fig. 2B). Furthermore, combined PARP inhibition and BRCA2 depletion further enhanced degradation of stalled forks (Fig. 2B). Surprisingly, however, when ATR and PARP were simultaneously inhibited, fork degradation was rescued (Fig. 2B). Notably, fork stabilization upon ATR inhibition was observed in both control‐depleted and BRCA2‐depleted cells (Fig. 2B). Interestingly, ATR inhibition did not prevent fork degradation in BRCA2‐depleted cells, in the absence of PARP inhibition, indicating that ATR inhibition does not rescue fork degradation *per se* (Fig. 2B). Of note, combined inhibition of PARP (using AZD‐2461) and ATR (using VE‐821 or VE‐822) in RPE‐1 cells resulted in similar findings (Fig. 2C), underscoring that inhibition of ATR does not exacerbate PARP inhibitor‐induced degradation of stalled replication forks.

Rather than further exacerbating replication‐born DNA lesions, ATR inhibition may promote premature mitotic entry in the presence of DNA lesions. To this end, BRCA2‐depleted HeLa cells were treated with olaparib and/or VE‐821 and stained for FANCD2 (Fanconi anemia group D2) and γ‐H2AX (Fig. 2D). Inhibition of ATR alone resulted in an increased amount of FANCD2 foci—but not γ‐H2AX foci—in prophase/pro‐metaphase cells (Fig. 2E,F). In contrast, PARP inhibition alone increased both γ‐H2AX and FANCD2 foci (Fig. 2E,F). Combined inhibition of ATR and PARP led to a significant further increase in γ‐H2AX and FANCD2 foci present in prophase/pro‐metaphase cells, with a larger effect size in BRCA2‐depleted cells compared to control‐depleted cells (Fig. 2E,F). Taken together, combined ATR and PARP inhibition results in premature mitotic entry in the presence of substantial amounts of DNA lesions.

### 3.4 ATR inhibition exacerbates PARP inhibitor‐induced mitotic aberrancies in BRCA2‐deficient cells

**Figure 3:**
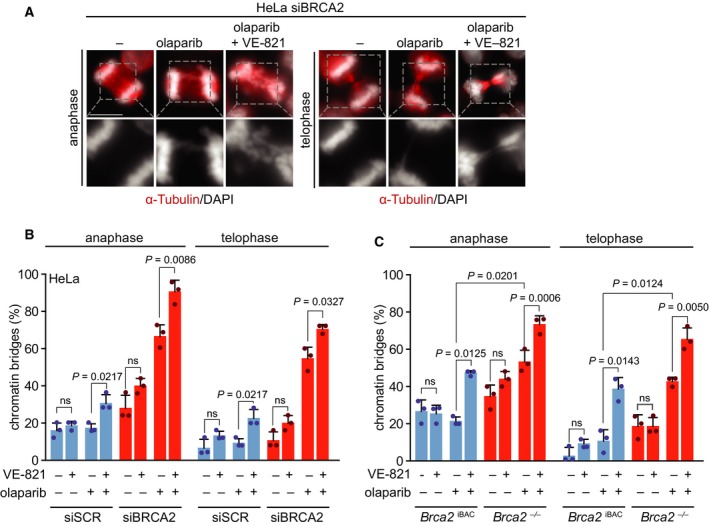
Combined PARP and ATR inhibition increases the amount of mitotic aberrancies. (A) HeLa cells were transfected with control (siSCR) or BRCA2 (siBRCA2 #1) siRNA for 24 h and treated with olaparib (0.5 μm) and/or VE‐821 (1 μm) for 24 h. Cells were fixed in formaldehyde (4%) and stained for α‐tubulin (red) and counterstained with DAPI (white). Representative immunofluorescence images are shown. Scale bar represents 10 µm. (B) Percentages of chromatin bridge‐positive cells (*n* = 25 events per condition, per experiment). Averages and standard deviations of three biological replicate experiments are shown. *P* values were calculated using two‐tailed Student’s *t*‐test. (C) *Brca2* ^iBAC^ cells and *Brca2* ^−/−^ cells were treated and analyzed as described for panel a. Averages and standard deviations of three biological replicate experiments are shown. *P* values were calculated using two‐tailed Student’s *t*‐test. Throughout the figure, ‘ns’ indicates not significant.

We previously reported that PARP inhibitor treatment of BRCA2‐depleted HeLa cells results in aberrant chromosome segregation (Schoonen *et al.*, 2017). In line with this notion, combined PARP and ATR inhibition was observed to result in increased mitotic defects and mitotic catastrophe (Kim *et al.*, 2017; Michelena *et al.*, 2018). However, how ATR inhibition contributes to this phenotype mechanistically remains elusive. To investigate whether premature mitotic entry upon ATR inhibition further increases mitotic aberrancies, we analyzed chromosome segregation defects. In line with previously published data (Schoonen *et al.*, 2017), BRCA2‐depleted cells showed increased numbers of anaphase chromatin bridges in response to PARP inhibition (67% in olaparib‐treated BRCA2‐depleted cells versus 17% in olaparib‐treated control cells) (Fig. 3A,B). Similarly, lagging chromosomes were more frequent (53% in olaparib‐treated BRCA2‐depleted cells versus 6% in olaparib‐treated control cells; Fig. S2B). Importantly, the majority of the anaphase chromatin bridges in BRCA2‐depleted cells remained unresolved (Fig. 3B).

Interestingly, when mitotic entry was accelerated in BRCA2‐depleted cells through ATR inhibition, chromatin bridge formation upon PARP inhibition was exacerbated in anaphase (91% versus 67%) and telophase (71% versus 55%) (Fig. 3B). Of note, combined PARP and ATR inhibition also increased the frequency of chromatin bridges as well as lagging chromosomes in control‐depleted cells (Fig. 3B and Fig. S2B). Similar mitotic defects were observed upon combined inhibition of PARP and ATR in *Brca2* ^−/−^ mammary tumor cells (Fig. 3C and Fig. S2C). Specifically, combined ATR and PARP inhibitor treatment in *Brca2* ^−/−^ cells increased chromatin bridges in anaphase (73% versus 53%) and telophase (65% versus 43%) and resulted in elevated levels of lagging chromosomes (63% versus 51%; Fig. 3C and Fig. S2C). Again, these effects were not limited to *Brca2* ^−/−^ cells, as HR‐proficient *Brca2* ^iBAC^ cells also showed increased chromatin bridge formation in anaphase (47% versus 21%) and telophase cells (39% versus 11%; Fig. 3C), as well as increased amounts of cells with lagging chromosomes (46% versus 19%; Fig. S2C). Although combined inhibition of ATR and PARP resulted in an elevation of chromatin bridges in both HR‐proficient and HR‐deficient cells, the effects were stronger in BRCA2‐depleted cells (Fig. 3B,C).

### 3.5 Delayed mitotic entry prevents mitotic aberrancies and genomic instability induced by combined ATR and PARP inhibition

**Figure 4:**
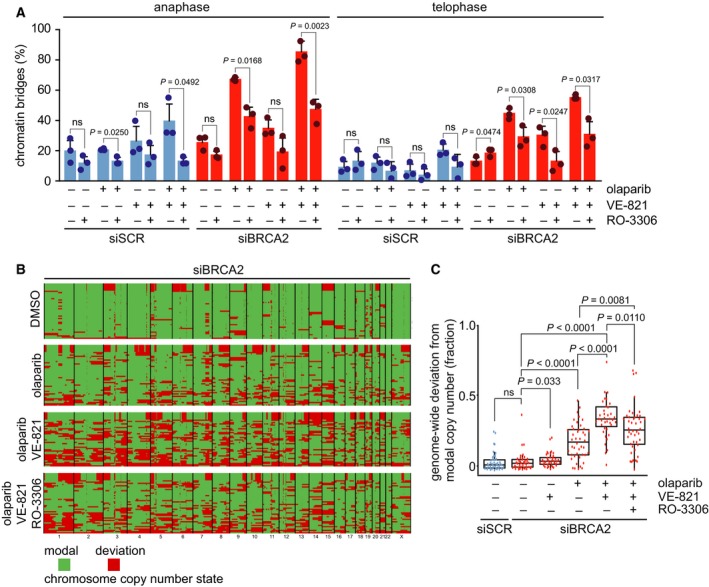
CDK1 inhibition prevents mitotic aberrancies and genomic instability induced by combined ATR and PARP inhibition. (A) HeLa cells were transfected with siSCR or siBRCA2 (siBRCA2 #1) for 24 h and were treated as indicated with olaparib (0.5 μm), VE‐821 (1 μm). Simultaneously, the CDK1 inhibitor RO‐3066 (10 μm) was added to cells for 24 h, to delay G2/M cell cycle transition. Subsequently, RO‐3066 was removed, and after 90 min, cells were fixed and stained for α‐tubulin (red) and counterstained with DAPI (white). Percentages of chromatin bridge‐positive cells (*n* = 15 events per condition, per experiment). Averages and standard deviations of three biological replicate experiments are shown. *P* values were calculated using two‐tailed Student’s *t*‐test. Throughout the figure, ‘ns’ indicates not significant. (B) HeLa cells were treated as in panel a and were harvested and frozen in medium containing 20% DMSO after 24 h. Cells were lysed and stained using Hoechst/PI, and single G1 nuclei were sorted. Genomic DNA was isolated from 46 single nuclei, and genomic libraries were included depending on library quality. Each row represents a single cell. Genome‐wide copy number plots were generated using the AneuFinder algorithm (see Section 2). Modal copy number states per ~ 1‐Mb bin are indicated: Green indicates modal copy number, whereas red indicates deviation from modal copy. Summary plots of indicated treatments are shown. Original ploidy scores are shown in Fig. S4. (C) Quantification of data from panel b, showing the fraction of bins per individual library deviating from the sample modal copy number. Statistical significance was determined using a Wilcoxon rank sum test (Mann–Whitney).

To corroborate that the increased formation of chromatin bridges upon ATR inhibition is due to premature mitotic entry, we delayed cell cycle progression at the G2/M transition through inhibition of CDK1. As expected, treatment with the CDK1 inhibitor RO‐3306 resulted in an accumulation of cells containing 4n DNA and adjourned mitotic entry (Fig. S3A,B). To test the effects of delayed mitotic entry, CDK1 was inhibited for 24 h, and cells were analyzed at 90 min after CDK1 inhibitor was washed out (Fig. 4A). Clearly, transient CDK1 inhibition reduced the percentage of PARP inhibitor‐induced chromatin bridges in BRCA2‐deficient cells in anaphase (43% versus 67%) as well as in telophase (29% versus 45%) (Fig. 4A), suggesting that PARP inhibitor‐induced DNA lesions are more efficiently resolved when mitotic entry is delayed. CDK1 inhibition caused the largest reduction in chromatin bridge formation in cells cotreated with PARP and ATR inhibitors (anaphase: 47% versus 86%; telophase: 31% versus 55%) (Fig. 4A). Notably, CDK1 inhibition also reduced the numbers of lagging chromosomes (Fig. S3C). Taken together, our findings show that ATR inhibition increases unresolved PARP inhibitor‐induced DNA lesions in mitosis, at least in part, due to accelerated mitotic entry.

Since combined inhibition of PARP and ATR induces anaphase chromatin bridges and lagging chromosomes (Fig. 3 and Fig. S2B,C), we next investigated the impact of this treatment on genome integrity. To this end, low‐coverage single‐cell whole‐genome sequencing (scWGS) was performed (Fig. S4) (Bakker *et al.*, 2016; van den Bos *et al.*, 2016). Control HeLa cells showed some degree of genomic instability, and BRCA2 depletion did not significantly exacerbate levels of genomic instability and ensuing heterogeneity within the time frame of this experimental setup (Fig. 4B,C). ATR inhibition alone led to minor elevation of genomic instability in BRCA2‐depleted cells, whereas olaparib treatment resulted in widespread focal copy number alterations (Fig. 4B,C). In line with our observation that combined ATR and PARP inhibition in BRCA2‐depleted cells led to persisting chromatin bridges (Fig. 3B), a significant increase in genomic instability was observed in these cells (Fig. 4B,C). Notably, CDK1 inhibition significantly reduced the levels of genomic instability, underlining that premature entry induced by ATR inhibition drives genomic instability in PARP inhibitor‐treated BRCA2‐depleted cancer cells.

### 3.6 ATR inhibition aggravates PARP inhibitor‐induced formation of cGAS‐positive micronuclei formation in BRCA2‐deficient cells

**Figure 5:**
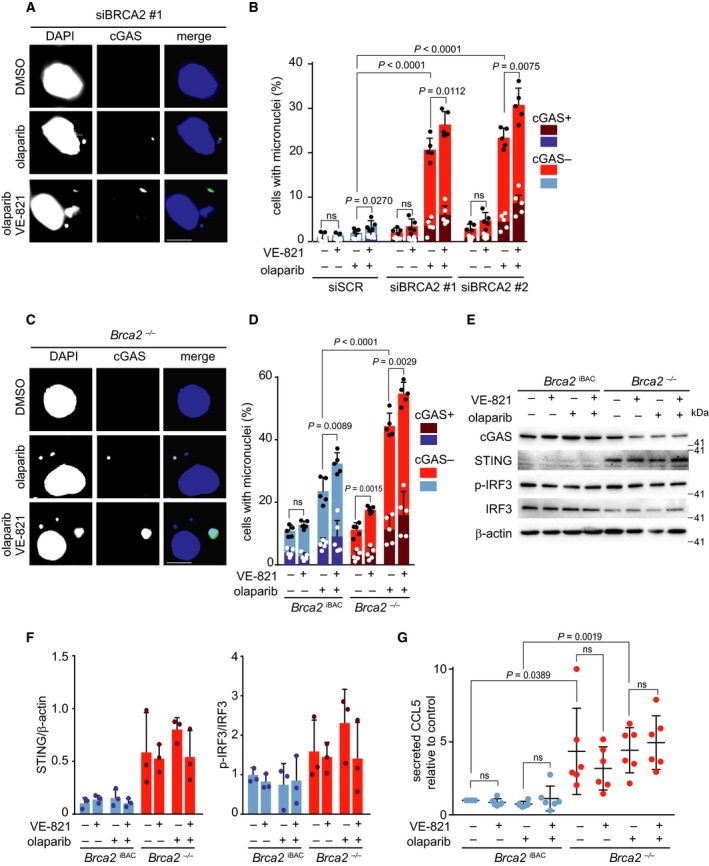
Combined ATR and PARP inhibition increases the amount of cGAS‐positive micronuclei. (A) HeLa cells were transfected with control siRNA (siSCR) or siRNA against BRCA2 (siBRCA2 #1, #HSS186121 siBRCA2 #2, #HSS101095) for 24 h and treated with olaparib (0.5 μm) and/or VE‐821 (1 μm) for 24 h. Cells were fixed in formaldehyde (4%) and stained for cGAS (green) and counterstained with DAPI (blue). Representative immunofluorescence images are presented. Scale bar represents 10 µm. (B) Percentages of cGAS‐positive and micronuclei‐positive cells (*n* = 200 events per condition per experiment). Averages and standard deviations of five biological replicate experiments are shown. *P* values were calculated using two‐tailed Student’s *t*‐test. (C) *Brca2* ^−/−^ and *Brca2* ^iBAC^ were treated with olaparib (0.5 μm) and/or VE‐821 (1 μm) for 24 h. Cells were fixed in formaldehyde (4%) and stained for cGAS (green) and counterstained with DAPI (blue). Representative immunofluorescence images are presented. Scale bar represents 10 µm. (D) Percentages of cGAS‐positive and micronuclei‐positive cells (*n* = 200 events per condition per experiment). Averages and standard deviations of five biological replicate experiments are shown. *P* values were calculated using two‐tailed Student’s *t*‐test. (E) *Brca2* ^−/−^ and *Brca2* ^iBAC^ were treated as described in panel c. Cell lysates were subsequently immunoblotted for cGAS, STING, phospho‐IRF3, IRF3, and β‐actin. (F) Quantification of data shown in panel e. Ratios of STING/β‐actin and p‐IRF3/IRF3 were normalized to controls. Averages and standard deviations of three biological replicate experiments are shown. *P* values were calculated using two‐tailed Student’s *t*‐test. (G) *Brca2* ^−/−^ and *Brca2* ^iBAC^ cells were treated as described in panel C, and the levels of CCL5 in media were determined. Data are normalized to *Brca2* ^iBAC^ and presented as averages and standard deviations of six biological replicate experiments. *P* values were calculated using two‐tailed Student’s *t*‐test. Throughout the figure, ‘ns’ indicates not significant.

The micronuclei that result from BRCA2 inactivation were recently shown to trigger a cGAS/STING‐dependent interferon response (Heijink *et al.*, 2019). We therefore assessed whether inhibition of ATR and PARP would exacerbate the formation of cGAS‐positive micronuclei and the cGAS/STING‐dependent interferon response. PARP inhibitor treatment resulted in increased formation of micronuclei in BRCA2‐depleted HeLa cells (20% in siBRCA2 #1 and 23% in siBRCA2 #2‐transfected olaparib‐treated HeLa cells versus 2% in olaparib‐treated control cells; Fig. 5A,B). Combined ATR and PARP inhibition further increased overall numbers of micronuclei as well as cGAS‐positive micronuclei in BRCA2‐depleted HeLa cells (*P* = 0.0112 and *P* = 0.0075, respectively; Fig. 5B). Detection of cytoplasmic DNA by cGAS leads to STING‐dependent interferon signaling, which includes upregulation of STING itself and the secretion of pro‐inflammatory cytokines (Ablasser *et al.*, 2013; Gao *et al.*, 2013; Sun *et al.*, 2013). In HeLa cells, BRCA2‐depletion did not alter STING expression levels nor levels of downstream components of cGAS/STING signaling (Fig. S5A), in line with viral HPV oncogenes in HeLa cells inactivating STING (Lau *et al.*, 2015). Therefore, we next assessed cGAS/STING activation upon PARP and ATR inhibition in *Brca2* ^−/−^ cells. Combined inhibition of ATR and PARP increased the overall numbers of micronuclei as well as the numbers of cGAS‐positive micronuclei in both *Brca2* ^−/−^ and *Brca2* ^iBAC^ cells (*P* = 0.0029 and *P* = 0.0089, respectively; Fig. 5C,D). In HR‐proficient *Brca2* ^IBAC^ cells, an increased number of micronuclei were observed upon PARP inhibition and combined ATR/PARP inhibition (Fig. 5D). However, a much larger increase was observed upon ATR and combined ATR/PARP inhibition in *Brca2* ^−/−^ cells (Fig. 5D). To assess whether the increase in cGAS‐positive micronuclei coincided with increased inflammatory signaling, we next assessed STING levels as well as phosphorylation of IRF3 (interferon regulatory factor 3) (Fig. 5E). We found a slight increase in p‐IRF3 in *Brca2* ^−/−^ cells compared to *Brca2* ^IBAC^ cells, however not when PARP and ATR inhibition were combined (Fig. 5E). Interestingly, STING was more abundant in untreated *Brca2* ^−/−^ cells compared to *Brca2* ^iBAC^ (Fig. 5F), possibly reflecting the consequences of HR deficiency of the *Brca2* ^−/−^ cells. To test whether the increased levels of cGAS‐positive micronuclei triggered a pro‐inflammatory cytokine response, we measured CCL5 (C‐C motif chemokine 5) levels in the supernatants of *Brca2* ^−/−^ and *Brca2* ^iBAC^ cells (Fig. 5G). Indeed, CCL5 production was elevated in *Brca2* ^−/−^ compared to *Brca2* ^iBAC^ (*P* = 0.0389). After 24 h of combined ATR and PARP inhibition, we observed increased CCL5 secretion, although this increase was not statistically significant (Fig. 5G). A more pronounced increase in CCL5 secretion was found after 72 h of combined inhibition of PARP and ATR (Fig. S5B). Taken together, these data show that combining PARP and ATR inhibitors leads to elevated numbers of micronuclei and increased CCL5 secretion.

## 4 Discussion

Although HR‐deficient cancer cells were shown to be profoundly sensitive to PARP inhibition, multiple mechanisms of acquired resistance have been described. Here, we show that ATR inhibition enhances the effect of PARP inhibitors in BRCA2‐deficient cells by accelerating entry into mitosis in the presence of DNA lesions. As a consequence, combined targeting of ATR and PARP leads to elevated levels of mitotic chromatin bridges, genomic instability, micronuclei formation, and cGAS/STING‐associated inflammatory signaling.

Our data confirm that PARP inhibition‐induced DNA damage can trigger G2/M checkpoint activation in HR‐deficient cancer cells (Jelinic and Levine, 2014; Maya‐Mendoza *et al.*, 2018; Prasad *et al.*, 2017; Ray Chaudhuri *et al.*, 2016; Rein *et al.*, 2015). Interestingly, we also observed a delayed mitotic entry upon olaparib treatment in HR‐proficient cells. Notably, using the nocodazole trap assay, *Brca2* ^iBAC^ cells showed pronounced G2 delay (Fig. S1E), whereas control‐transfected HeLa cells did not show delayed G2/M progression (Fig. 1A). Likely, the degree to which cells delay G2 progression upon PARP inhibition differs between cell lines. Indeed, the moderate but reproducible delay in mitotic entry in HeLa cells is only noticeable in a time‐course analysis of synchronized cells (Fig. 1E and Fig. S1E), but not using endpoint assays (Fig. 1A and Fig. S1C).

ATR inhibition was previously shown to sensitize cancer cells to various DNA‐damaging agents (Abu‐Sanad *et al.*, 2015; Jossé *et al.*, 2014; Reaper *et al.*, 2011), including synergistic interactions between PARP and ATR inhibition in HR‐deficient tumors (Huntoon *et al.*, 2013; Kim *et al.*, 2017). We show here that ATR inhibition sensitizes HR‐deficient cells to PARP inhibition by forcing premature mitotic entry in the presence of DNA lesions. Specifically, we observed an increase in mitotic FANCD2 foci, a proxy for under‐replicated DNA (Chan *et al.*, 2009). These results are in agreement with previous findings that ATR regulates the S‐G2 transition and that ATR inhibition results in under‐replicated DNA (Saldivar *et al.*, 2017). We reinstated a G2/M delay through blockade of CDK1, which rescued the effects of ATR inhibition on genomic stability in PARP‐inhibited BRCA2‐depleted cells, reinforcing the role of ATR as a cell cycle checkpoint kinase.

ATR also controls additional mechanisms, which could contribute to the potentiating effects of ATR inhibition toward PARP inhibitor‐mediated cytotoxicity in BRCA2‐defective cells. For example, ATR inhibition has been shown to sensitize PARP inhibitor‐resistant *BRCA1* mutant cancer cells to PARP inhibition through blocking protection of stalled replication forks (Yazinski *et al.*, 2017). However, we found that increased fork degradation was not associated with elevated levels of cell death. This finding is in accordance with recent observations (Feng and Jasin, 2017) and may be explained by a role for ATR/CHK1 signaling in preventing EMI1‐mediated degradation of RAD51 (Marzio *et al.*, 2018). Specifically, RAD51 has been shown to be required for reversal of stalled forks, an essential step in fork degradation (Mijic *et al.*, 2017). Inhibition of ATR may lead to unscheduled RAD51 degradation (Marzio *et al.*, 2018). Although ATR inhibitor‐mediated RAD51 destabilization will lead to a further decrease in HR DNA repair in BRCA‐mutant cells, RAD51 destabilization could prevent fork degradation, which would explain our observation that ATR inhibition rescues fork degradation upon PARP inhibition in BRCA2‐defective cells. Alternatively, the rescue of fork degradation upon combined ATR and PARP inhibition could be explained by a requirement for ATR in the proper localization or activation of nucleases that target stalled replication forks, including MRE11 (meiotic recombination 11), MUS81 (crossover junction endonuclease MUS81), and DNA2 (DNA replication ATP‐dependent helicase/nuclease DNA2) (Ray Chaudhuri *et al.*, 2016; Rondinelli *et al.*, 2017; Xu *et al.*, 2017). Furthermore, it was recently shown that loss of p53 results in altered origin firing and differential responses replication stress (Benedict *et al.*, 2018), which may underlie the observed differences in fork stability between HeLa and RPE‐1 cells upon ATR inhibition.

Cytosolic DNA as a result of genomic instability has recently been reported to trigger a cGAS/STING‐dependent interferon response (MacKenzie *et al.*, 2017). In line with this notion, loss of BRCA2 has also been reported to activate cGAS/STING (Heijink *et al.*, 2019). We observed that treatment of BRCA2‐defective cells with PARP inhibitor increases mitotic defects and results in elevated numbers of micronuclei. Importantly, the numbers of micronuclei, including cGAS‐positive micronuclei, increased upon ATR inhibition. Recently, cytosolic DNA induced by olaparib treatment in BRCA1‐deficient tumors was shown to render these tumors sensitive to PD‐1 blockade treatment (Ding *et al.*, 2018). Moreover, antitumor effects of PARP inhibitor treatment in BRCA‐deficient triple‐negative breast cancers were shown to depend on cGAS/STING and subsequent T‐cell responses (Pantelidou *et al.*, 2019). Based on our data, combining ATR and PARP inhibition could be used to further increase micronuclei formation and thereby enforce the subsequent cGAS/STING‐mediated inflammatory response. Such combination treatment may be useful in potentiating sensitivity toward immune checkpoint inhibitor treatment.

## 5 Conclusions

**Figure 6:**
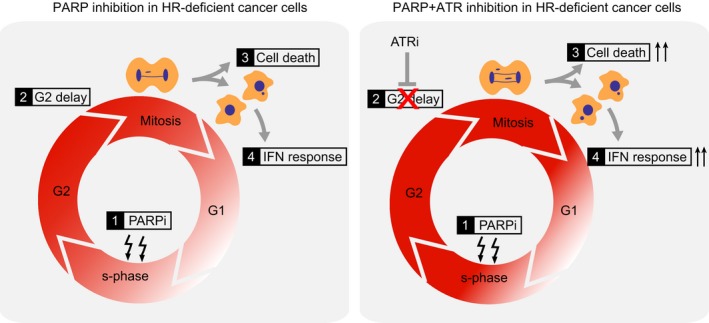
Model depicting how combined inhibition of ATR and PARP in HR‐deficient cells causes premature mitotic entry with ensuing mitotic aberrancies, increased interferon responses, and cell death.

Our data show that PARP inhibitor‐induced replication lesions that are not resolved in a timely fashion can lead to mitotic entry in the presence of DNA lesions and ensuing mitotic aberrancies and cell death (Fig. 6). Targeting the cell cycle checkpoint kinase ATR can be utilized to induce premature mitotic entry and thereby increase the cytotoxicity of PARP inhibitors in HR‐deficient tumor cells. Further research is warranted to extrapolate these results to other cell cycle checkpoint components (e.g., CHK1 and WEE1), and to explore these combination treatments in order to provoke inflammatory signaling, a key determinant of response to immune checkpoint inhibitors.

## Conflict of interest

The authors declare no conflict of interest.

## Author contributions

PMS, YPK, and MATMvV conceived the project, analyzed the data, and wrote the manuscript. PMS, YPK, and EW performed experiments. BB and FF analyzed single‐cell sequencing data. DCJS performed single‐cell sequencing.

## Supporting information


**Fig. S1.** PARP inhibition induces a dose‐dependent G2‐arrest, which is abrogated by ATR inhibition. (A) HeLa cells were transfected with control or BRCA2 (siBRCA2 #1’) siRNAs for 24 hours and subsequently treated with DMSO, olaparib (1, 5, or 10 µM) 24 hours prior to harvesting. Next, cells were treated with nocodazole (250 ng/ml) for 18 hours. DNA content (propidium iodine) and pH3‐Ser10/Alexa‐647 were assessed by flow cytometry on a Becton Dickinson FACSCalibur (Becton Dickinson, Franklin Lakes, NJ, USA). A minimum of 10,000 events were analyzed per sample. Averages and standard deviations of 3 biological replicates are shown (n = 3). *P* values were calculated using two‐tailed Student’s t‐test. (B) RNA of *Brca2* ^+/+^, *Brca2* ^−/−^, and *Brca2* ^iBAC^ cells was isolated and RT‐PCR was performed using oligos directed to *Brca2* exons 10‐11, *Brca2* exons 18‐19, *Hprt*, or *Gapdh*. (C)* Brca2* ^−/−^ and *Brca2* ^iBAC^ cells were irradiated (5 Gy) and fixed in formaldehyde (4%) after 4 hours. Subsequently, cells were stained for γ‐H2AX (red) and RAD51 (green) and counterstained with DAPI (blue). Scale bar represents 10 µm. (D) *Brca2* ^−/−^ and *Brca2* ^iBAC ^cells were treated and analyzed as described in panel A. (E/F) HeLa cells were transfected with siBRCA2 or siSCR for 24 hours and subsequently incubated with thymidine (2 mM) for 17 hours. Cells were then released for 9 hours in prewarmed growth media and again treated for 17 hours with thymidine prior to release in growth media supplemented with DMSO, olaparib (10 µM), and/or VE‐821 (1 µM). Cells were harvested at the indicated time points. Phospho‐Ser10‐histone‐H3/Alexa‐488 was assessed by flow cytometry on a Becton Dickinson FACSCalibur (Becton Dickinson, Franklin Lakes, NJ, USA). A minimum of 10,000 events were analyzed per sample. (G) HeLa cells were transfected with control siRNA (#12935300) or BRCA2 siRNA (siBRCA2 #2) and were treated with the indicated concentrations of olaparib and/or VE‐821. Methyl‐thiazol tetrazolium (MTT) was added (final concentration: 0.5 mg/mL) for 4 hours, and viability was assessed by colorimetric measurement. (H) Combination indices were determined using CompuSyn software.

**Fig. S2.** Combined PARP and ATR inhibition increases the amount of lagging chromosomes. (A) HeLa cells were transfected with control siRNAs (‘siSCR’, #12935300) or siRNAs targeting BRCA2 (‘siBRCA2 #1’ ‘siBRCA2 #2’) for 24 hours, and were next treated with PARP inhibitor olaparib (1 μM) for 24 hours. Cell lysates were subsequently immunoblotted for BRCA2, poly‐(ADP‐ribose) polymers (PAR), phospho‐ATR, and β‐actin. (B) HeLa cells were transfected with control siRNA (#12935300) or BRCA2 siRNA (siBRCA2 #1) for 24 hours and treated with olaparib (0.5 µM) and/or VE‐821 (1 µM), cells with lagging chromosomes (panel c, n = 50 events per condition, per experiment) were quantified. Averages and standard deviations of 3 biological replicate experiments are shown. *P* values were calculated using two‐tailed Student’s t‐test. (C) *Brca2* ^−/−^ and *Brca2* ^iBAC^ cells were treated with olaparib (0.5 µM) and/or VE‐821 (1 µM), cells with lagging chromosomes (n = 50 events per condition per experiment) were quantified. Averages and standard deviations of 3 biological replicate experiments are shown. *P* values were calculated using two‐tailed Student’s t‐test.

**Fig. S3.** CDK1 inhibition prevents induction of lagging chromosomes upon combined PARP and ATR inhibition. (A/B) HeLa cells were transfected with siSCR or siBRCA2 for 24 hours, and were subsequently treated with the CDK1 inhibitor RO‐3066 (10 μM) for 24 hours. RO‐3066 was removed, and cells were fixed after 90 minutes. DNA content (propidium iodine) and MPM‐2/Alexa‐647 positivity were assessed by flow cytometry on a Becton Dickinson FACSCalibur (Becton Dickinson, Franklin Lakes, NJ, USA). A minimum of 10,000 events were analyzed per sample. (C) HeLa cells were transfected with siSCR or siBRCA2 (siBRCA2 #1) for 24 hours and were treated with as indicated with olaparib (0.5 μM), VE‐821 (1 μM). Simultaneously, the CDK1 inhibitor RO‐3066 (10 μM) was added to cells for 24 hours, to delay G2/M cell cycle transition. Subsequently, RO‐3066 was removed and after 90 minutes, cells were fixed and stained for α‐tubulin (red) and counterstained with DAPI (white). Percentages of lagging chromosomes cells (n = 30 events per condition, per experiment). Averages and standard deviations of 3 biological replicate experiments are shown. *P* values were calculated using two‐tailed Student’s t‐test. Throughout the figure, ‘ns’ indicates not significant.

**Fig. S4.** CDK1 inhibition rescues genomic instability induced by combined ATR and PARP inhibition. HeLa cells were transfected with siSCR or siBRCA2 for 24 hours, and were subsequently treated with DMSO, olaparib (0.5 µM), VE‐821 (1 µM), and/or RO‐3306 (10 µM) as indicated for 24 hours. Cells were subsequently harvested and frozen in medium containing 20% DMSO. Cells were lysed and stained using Hoechst/PI, and single G1 nuclei were sorted. Genomic DNA was isolated of 46 single nuclei per condition, and resulting genomic libraries were included depending on library quality. Every row represents a single cell. Genome‐wide copy number plots were generated using the AneuFinder algorithm (see Materials and Methods). Copy number states were calculated for ~1‐Mb bins, and depicted by color coding.

**Fig. S5.** Combined ATR and PARP inhibition increases secretion of CCL5. (A) HeLa cells were transfected with control siRNAs (‘siSCR’, #12935300) or siRNAs targeting BRCA2 (‘siBRCA2 #1’ or ‘siBRCA2 #2’) for 48 hours. Cell lysates were subsequently immunoblotted for cGAS, STING, p‐IRF3, IRF3, and β‐actin. (B) *Brca2* ^−/−^ and *Brca2* ^iBAC^ cells were treated with olaparib (0.5 µM), VE‐821 (1 µM) for 24, 48 or 72 hours and levels of CCL5 in media were determined. Data are normalized to 1000 cells.
