# Supplementary figures and images for: Premature mitotic entry induced by ATR inhibition potentiates olaparib inhibition‐mediated genomic instability, inflammatory signaling, and cytotoxicity in BRCA2‐deficient cancer cells
Source: Mol Oncol. 2019 Oct 21;13(11):2422–40. doi: 10.1002/1878-0261.12573 (PMC6822251; doi:10.1002/1878-0261.12573)

## Supplemental Figure S1

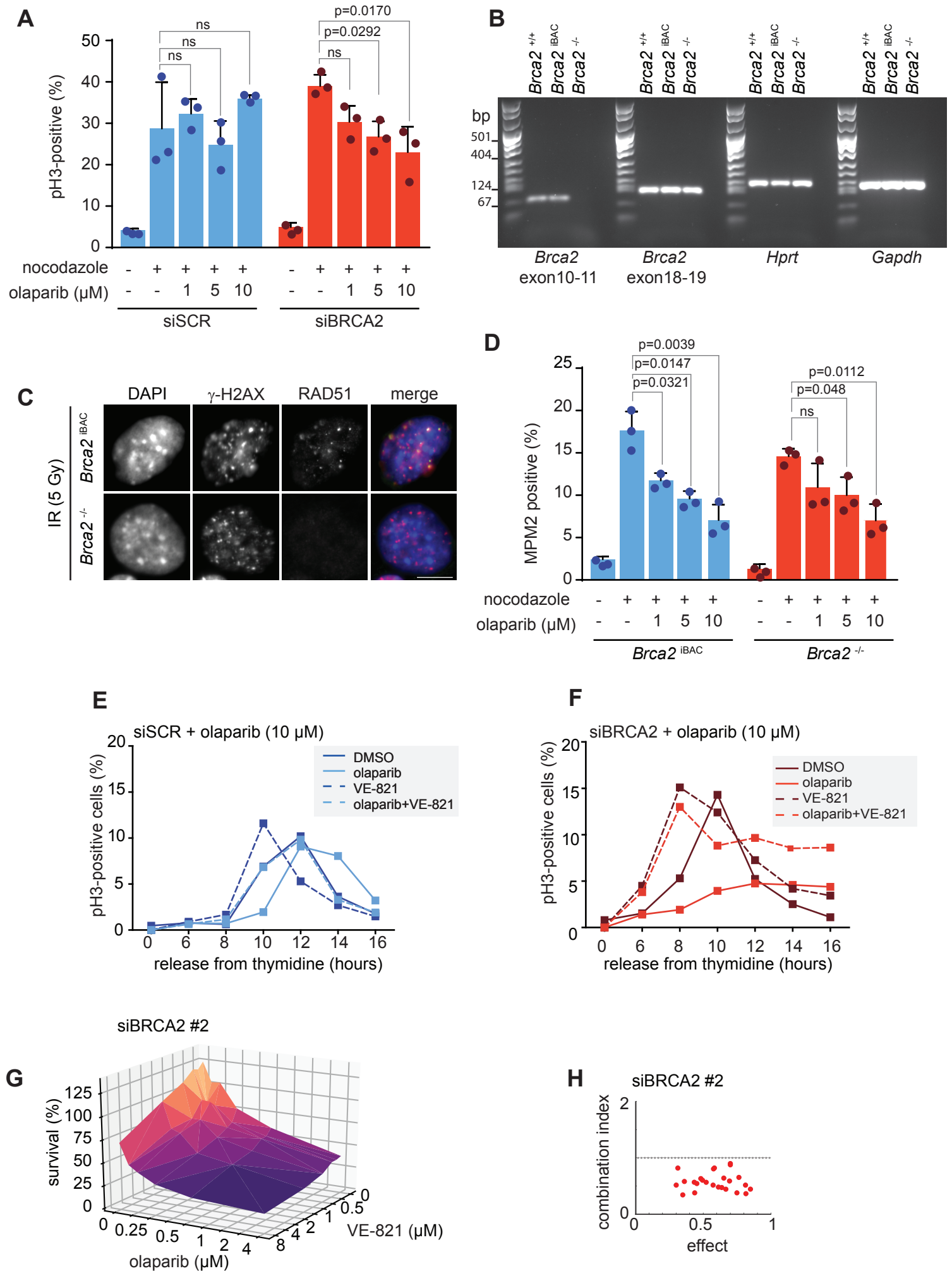

Supplement: Supplementary file 1 — Fig. S1. PARP inhibition induces a dose‐dependent G2‐arrest, which is abrogated by ATR inhibition. (A) HeLa cells were transfected with control or BRCA2 (siBRCA2 #1’) siRNAs for 24 hours and subsequently treated with DMSO, olaparib (1, 5, or 10 µM) 24 hours prior to harvesting. Next, cells were treated with nocodazole (250 ng/ml) for 18 hours. DNA content (propidium iodine) and pH3‐Ser10/Alexa‐647 were assessed by flow cytometry on a Becton Dickinson FACSCalibur (Becton Dickinson, Franklin Lakes, NJ, USA). A minimum of 10,000 events were analyzed per sample. Averages and standard deviations of 3 biological replicates are shown (n = 3). P values were calculated using two‐tailed Student’s t‐test. (B) RNA of Brca2 +/+, Brca2 −/−, and Brca2 iBAC cells was isolated and RT‐PCR was performed using oligos directed to Brca2 exons 10‐11, Brca2 exons 18‐19, Hprt, or Gapdh. (C) Brca2 −/− and Brca2 iBAC cells were irradiated (5 Gy) and fixed in formaldehyde (4%) after 4 hours. Subsequently, cells were stained for γ‐H2AX (red) and RAD51 (green) and counterstained with DAPI (blue). Scale bar represents 10 µm. (D) Brca2 −/− and Brca2 iBAC cells were treated and analyzed as described in panel A. (E/F) HeLa cells were transfected with siBRCA2 or siSCR for 24 hours and subsequently incubated with thymidine (2 mM) for 17 hours. Cells were then released for 9 hours in prewarmed growth media and again treated for 17 hours with thymidine prior to release in growth media supplemented with DMSO, olaparib (10 µM), and/or VE‐821 (1 µM). Cells were harvested at the indicated time points. Phospho‐Ser10‐histone‐H3/Alexa‐488 was assessed by flow cytometry on a Becton Dickinson FACSCalibur (Becton Dickinson, Franklin Lakes, NJ, USA). A minimum of 10,000 events were analyzed per sample. (G) HeLa cells were transfected with control siRNA (#12935300) or BRCA2 siRNA (siBRCA2 #2) and were treated with the indicated concentrations of olaparib and/or VE‐821. Methyl‐thiazol tetrazolium (MTT) was added (fi [file MOL2-13-2422-s001.pdf]

Supplemental Figure S2

A

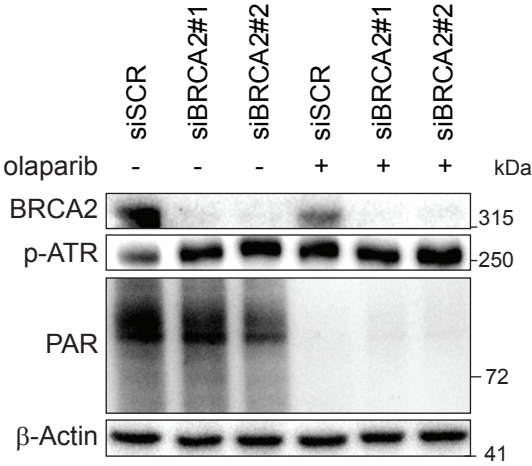

B

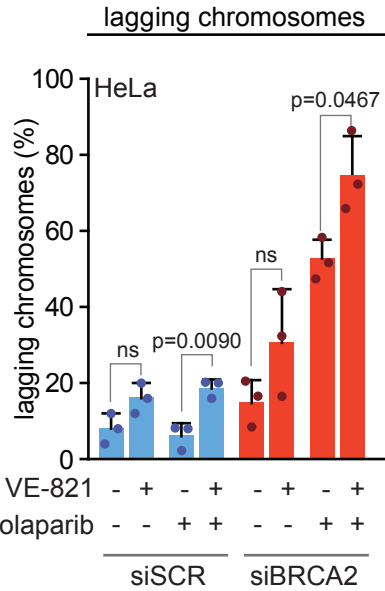

C

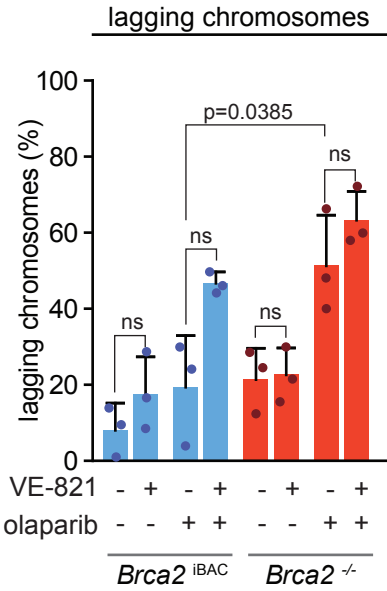

Supplement: Supplementary file 2 — Fig. S2. Combined PARP and ATR inhibition increases the amount of lagging chromosomes. (A) HeLa cells were transfected with control siRNAs (‘siSCR’, #12935300) or siRNAs targeting BRCA2 (‘siBRCA2 #1’ ‘siBRCA2 #2’) for 24 hours, and were next treated with PARP inhibitor olaparib (1 μM) for 24 hours. Cell lysates were subsequently immunoblotted for BRCA2, poly‐(ADP‐ribose) polymers (PAR), phospho‐ATR, and β‐actin. (B) HeLa cells were transfected with control siRNA (#12935300) or BRCA2 siRNA (siBRCA2 #1) for 24 hours and treated with olaparib (0.5 µM) and/or VE‐821 (1 µM), cells with lagging chromosomes (panel c, n = 50 events per condition, per experiment) were quantified. Averages and standard deviations of 3 biological replicate experiments are shown. P values were calculated using two‐tailed Student’s t‐test. (C) Brca2 −/− and Brca2 iBAC cells were treated with olaparib (0.5 µM) and/or VE‐821 (1 µM), cells with lagging chromosomes (n = 50 events per condition per experiment) were quantified. Averages and standard deviations of 3 biological replicate experiments are shown. P values were calculated using two‐tailed Student’s t‐test. [file MOL2-13-2422-s002.pdf]

Supplemental Figure S3

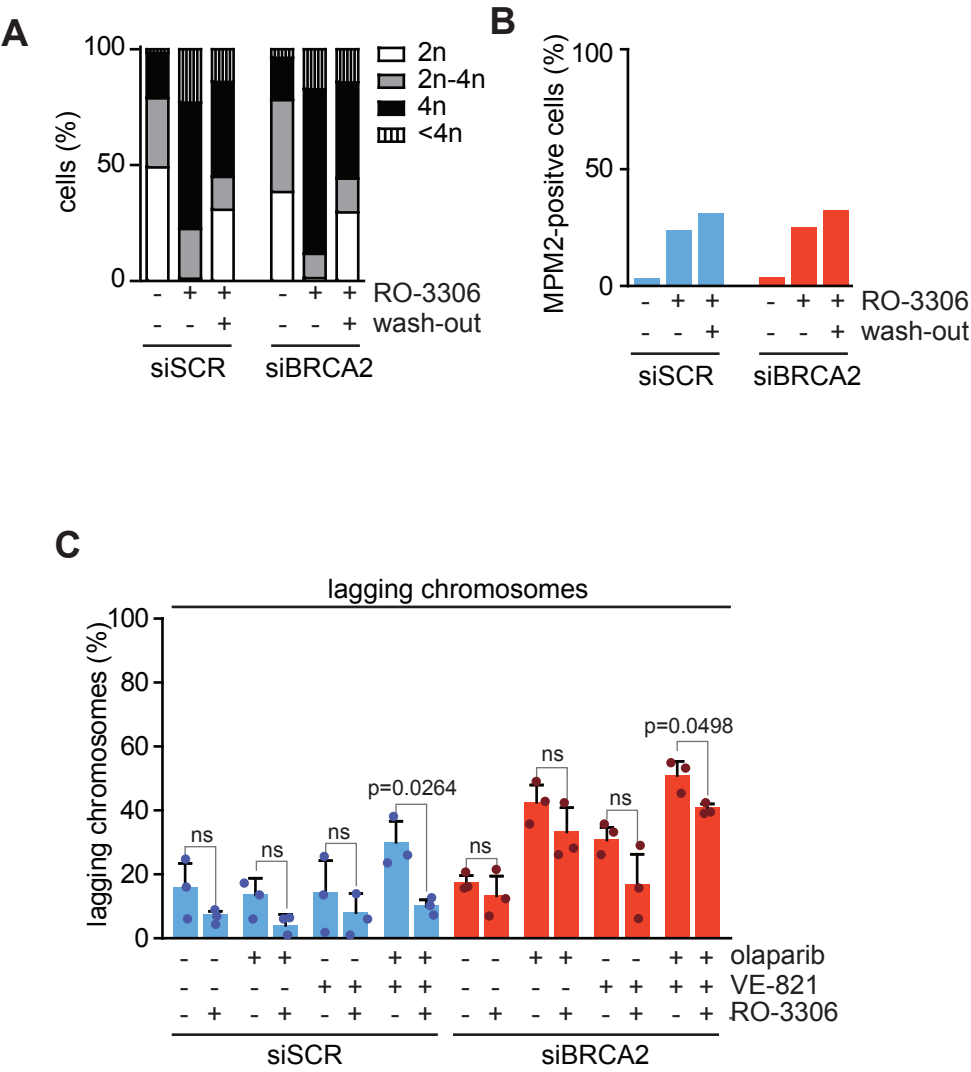

Supplement: Supplementary file 3 — Fig. S3. CDK1 inhibition prevents induction of lagging chromosomes upon combined PARP and ATR inhibition. (A/B) HeLa cells were transfected with siSCR or siBRCA2 for 24 hours, and were subsequently treated with the CDK1 inhibitor RO‐3066 (10 μM) for 24 hours. RO‐3066 was removed, and cells were fixed after 90 minutes. DNA content (propidium iodine) and MPM‐2/Alexa‐647 positivity were assessed by flow cytometry on a Becton Dickinson FACSCalibur (Becton Dickinson, Franklin Lakes, NJ, USA). A minimum of 10,000 events were analyzed per sample. (C) HeLa cells were transfected with siSCR or siBRCA2 (siBRCA2 #1) for 24 hours and were treated with as indicated with olaparib (0.5 μM), VE‐821 (1 μM). Simultaneously, the CDK1 inhibitor RO‐3066 (10 μM) was added to cells for 24 hours, to delay G2/M cell cycle transition. Subsequently, RO‐3066 was removed and after 90 minutes, cells were fixed and stained for α‐tubulin (red) and counterstained with DAPI (white). Percentages of lagging chromosomes cells (n = 30 events per condition, per experiment). Averages and standard deviations of 3 biological replicate experiments are shown. P values were calculated using two‐tailed Student’s t‐test. Throughout the figure, ‘ns’ indicates not significant. [file MOL2-13-2422-s003.pdf]

Supplemental Figure S4

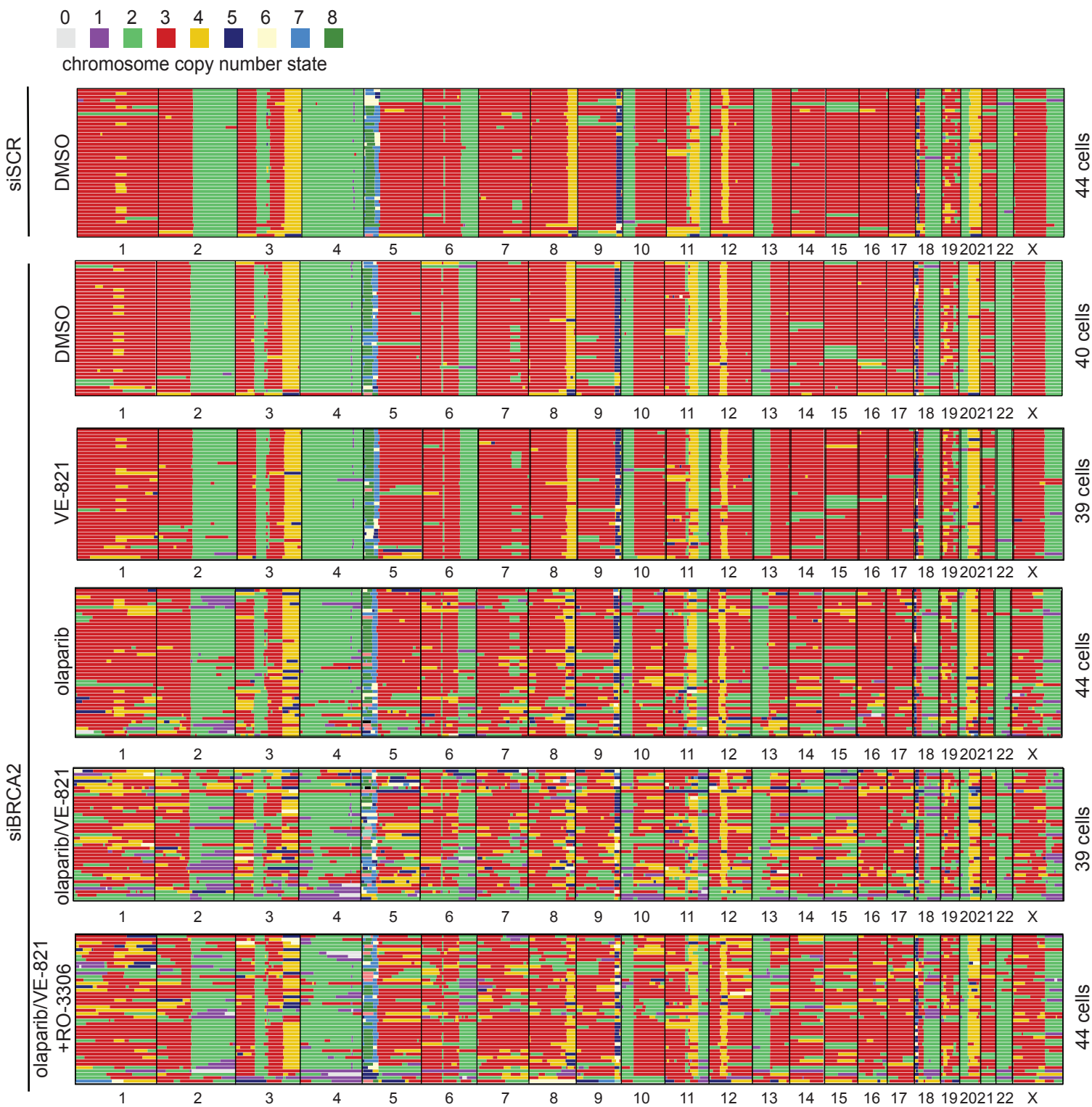

Supplement: Supplementary file 4 — Fig. S4. CDK1 inhibition rescues genomic instability induced by combined ATR and PARP inhibition. HeLa cells were transfected with siSCR or siBRCA2 for 24 hours, and were subsequently treated with DMSO, olaparib (0.5 µM), VE‐821 (1 µM), and/or RO‐3306 (10 µM) as indicated for 24 hours. Cells were subsequently harvested and frozen in medium containing 20% DMSO. Cells were lysed and stained using Hoechst/PI, and single G1 nuclei were sorted. Genomic DNA was isolated of 46 single nuclei per condition, and resulting genomic libraries were included depending on library quality. Every row represents a single cell. Genome‐wide copy number plots were generated using the AneuFinder algorithm (see Materials and Methods). Copy number states were calculated for ~1‐Mb bins, and depicted by color coding. [file MOL2-13-2422-s004.pdf]

Supplemental Figure S5

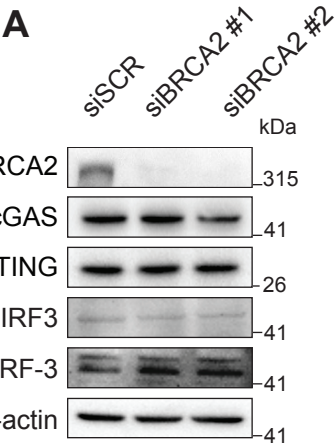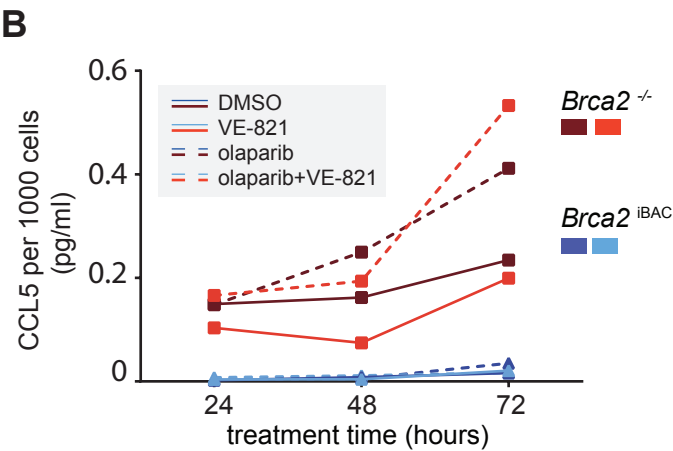

Supplement: Supplementary file 5 — Fig. S5. Combined ATR and PARP inhibition increases secretion of CCL5. (A) HeLa cells were transfected with control siRNAs (‘siSCR’, #12935300) or siRNAs targeting BRCA2 (‘siBRCA2 #1’ or ‘siBRCA2 #2’) for 48 hours. Cell lysates were subsequently immunoblotted for cGAS, STING, p‐IRF3, IRF3, and β‐actin. (B) Brca2 −/− and Brca2 iBAC cells were treated with olaparib (0.5 µM), VE‐821 (1 µM) for 24, 48 or 72 hours and levels of CCL5 in media were determined. Data are normalized to 1000 cells. [file MOL2-13-2422-s005.pdf]
